# Relative mobility of the pelvis and spine during trunk axial rotation in chronic low back pain patients: A case-control study

**DOI:** 10.1371/journal.pone.0186369

**Published:** 2017-10-17

**Authors:** Masashi Taniguchi, Hiroshige Tateuchi, Satoko Ibuki, Noriaki Ichihashi

**Affiliations:** 1 Division of Physical Therapy, Rehabilitation Units, Shiga University of Medical Science Hospital, Otsu, Shiga, Japan; 2 Department of Physical Therapy, Graduate School of Medicine, Kyoto University, Kyoto-City, Kyoto, Japan; University of Potsdam, GERMANY

## Abstract

**Background:**

Trunk axial rotation is a risk factor for chronic low back pain (CLBP). The characteristics of rotational mobility in the pelvis and spine among CLBP patients are not fully understood.

**Purpose:**

The purpose of this study was to examine three-dimensional kinematic changes, and to compare the differences of rotational mobility and coupled motion, in patients with and without CLBP.

**Methods:**

Fifteen patients with CLBP and 15 age and sex matched healthy subjects participated in this study. Each subject performed trunk rotation to maximum range of motion (ROM) in a standing position. The kinematics data was collected using a three-dimensional motion analysis system. The outcomes measured were the rotational ROM and the spine/pelvis ratio (SPR) in transvers plane at both maximum and 50% rotation position. The coupled angles in sagittal and frontal planes were also measured.

**Results:**

No significant differences in rotational ROM of the thorax, pelvis, and spine were observed between two groups at maximum rotation position. However, there was a significant interaction between groups and rotational ROM of pelvis and spine (F = 4.57, p = 0.04), and the SPR in CLBP patients was significantly greater than that of the healthy subjects (CLBP; 0.50 ± 0.10 Control; 0.41 ± 0.12, p = 0.04). The results at 50% rotation position were similar to that at maximum rotation. This indicates a relative increase in spinal rotation in the CLBP patients during trunk rotation. Moreover, the CLBP patients exhibited a significantly higher anterior tilt of the pelvis and extension of the spine in the sagittal plane coupled with rotation.

**Conclusions:**

CLBP patients had relative hyper rotational mobility of the spine as well as excessive spinal extension coupled with trunk rotation. These results suggest that uncoordinated trunk rotation might be a functional failure associated with CLBP.

## Introduction

Chronic low back pain (CLBP) has been described as a major health problem regardless of generation [1]. Many cases of low back pain are related to job tasks that involve lifting and trunk axial rotation (trunk rotation) [2, 3]. Previous studies have reported that motion involving trunk rotation increase the risk of low back pain by 1.51–2.28 times compared with subjects who do not perform trunk rotation, and is also a risk factor for its recurrence and chronicity [4, 5]. Although these studies investigated the frequency and duration of trunk rotation as a prospective cohort study, kinematics has not been evaluated. Studying the kinematics of trunk rotation in CLBP patients is important for estimating the mechanical stress to the spine.

Trunk rotation mainly is achieved by rotation of the pelvis (in other words, by rotation of both the hip joints), with a small contribution from the spine [6]. Rotational mobility in the transverse plane of the lumbar spine is small, compared with that of the thoracic spine [7]. Excessive spinal rotation at the end of range of motion (ROM) may be associated with an increased risk of CLBP, therefore, it is necessary to examine the kinematic characteristics in patients with CLBP. A previous study [8] investigated rotational ROM in the spine during maximum trunk rotation using a three-dimensional motion analysis system, and indicated that the lumber spine rotation in CLBP patients was significantly greater compared with control subjects. On the other hand, Al-Eisa et al, [9] has reported that CLBP patients have movement asymmetry, though there were no difference in rotation ROM in the lumber spine during rotation task. The onset of pain occurs at approximately half of the maximum rotational ROM during activities of daily living in subjects with CLBP [10]. Although some previous studies [8, 9] have investigated the kinematic differences between subjects with and without CLBP during trunk rotation, the characteristics of rotational mobility still are not fully understood. Especially, there is no information regarding analysis of the movement pattern focusing on the coordination between pelvis and spine mobility in CLBP patients during trunk rotation.

It is known that coupled motion, or coupling biomechanics—the rotation or translation of a vertebral body about or along 1 axis that is consistently associated with the main rotation or translation about another axis [11]—exists in spine motion. Previous studies [12, 13] have reported that excessive translation of a vertebral body in the sagittal and/or frontal plane during rotational movement can lead to spinal disorders due to increased stress on the intervertebral discs or joints. However, Sung et al, [8] examined the coupled motion of the lumbar spine in patients with CLBP using a three-dimensional motion analysis system, and showed that there was no difference compared with healthy subjects. Thus, there is no consensus on the kinematic characteristics of coupled motion in patients with CLBP. Since the CLBP patients exhibited significantly higher coupled rotation during trunk lateral flexion [9, 14], the coupled motion in sagittal and frontal plane during trunk rotation may also have the same pattern. Thus, evaluation of coupled motion during trunk rotation is necessary while determining the kinematic characteristics of CLBP patients.

The purpose of the present study was to examine the three-dimensional kinematic difference during trunk rotation, and to compare the rotational mobility and coupled motion in patients with and without CLBP. We hypothesized that CLBP patients 1) differ in movement pattern rather than in maximum rotation ROM in the transverse plane, and 2) have excessive spinal motion coupled with rotation, compared with healthy subjects. Understanding this would provide an insight into the kinematic characteristics of CLBP patients (in particular, their spine-pelvis coordination strategies), and would help to develop rehabilitation programs.

## Materials and methods

Fifteen patients with nonspecific CLBP and 15 age and sex matched-healthy control subjects volunteered for this study (Table 1). All the subjects were students at Kyoto University and played sports activities at a recreational level. The inclusion criteria for the CLBP group were as follows: history of LBP with an intensity greater than 30 mm on the visual analog scale (VAS), continuous pain for >3 months, and ability to perform the motion task. CLBP patients were further screened using a novel questionnaire (S1 and S2 Tables), and those having pain in the lumbosacral region, buttocks, and thighs, with no nerve root symptoms, were carefully selected. The items of questionnaire included the kind, degree, and duration of LBP; the type of motion that induce LBP; whether any medical attention was sought; and complications. The patients who had not sought any medical attention for their LBP were examined by an orthopaedic surgeon to check whether they met the inclusion criteria. Exclusion criteria for the CLBP patients included previous lumbar surgery, history of neuromuscular or joint disease, and motor signs of nerve root compression. A total of 18 patients were screened using the questionnaire, of which 3 patients who had a diagnosis of disc herniation in the past were excluded. Seven patients who had not previously sought medical attention met the inclusion criteria, and were included in the study. Finally, 15 patients were included in CLBP group. The mean scores on the VAS and Oswestry Disability Index (ODI) in the CLBP group within the month prior to experimental period are presented in Table 1. Subjects in the control group were included if they had no history of LBP that continued for >3 months in their lifetime, no LBP in the month prior to experimental period. All subjects were informed of the purpose of the study, and they provided written informed consent before participating. All test protocols were approved by the Ethics Committee of Kyoto University (approval no.E-875).

**Table 1:** Summary of subject anthropometrics.

	CLBP patients	Controls	*P*-value
	mean ± SD	mean ± SD	
Sex (male/female)	9/6	9/6	1.00
Age (years)	23.0 ± 2.2	25.1 ± 5.2	0.17
[range]	[20–37]	[21–28]	
Height (cm)	167.8 ± 8.9	166.3 ± 8.5	0.64
Weight (kg)	59.8 ± 8.2	58.9 ± 7.7	0.77
Right handed (%)	100	100	1.00
Sports activity with rotational motion (n)	5	4	1.00
VAS (mm)	34.9 ± 24.2	0	N/A
ODI (%)	14.8 ± 10.9	―	N/A
Duration of CLBP (months)	18.8 ± 21.9	none	N/A
LBP with trunk rotation (n)	11	none	N/A

SD: standard deviation; CLBP: chronic low back pain; VAS: visual analog scale; ODI: oswestry disability index. N/A indicates not applicable.

### The motion task

**Fig 1:**
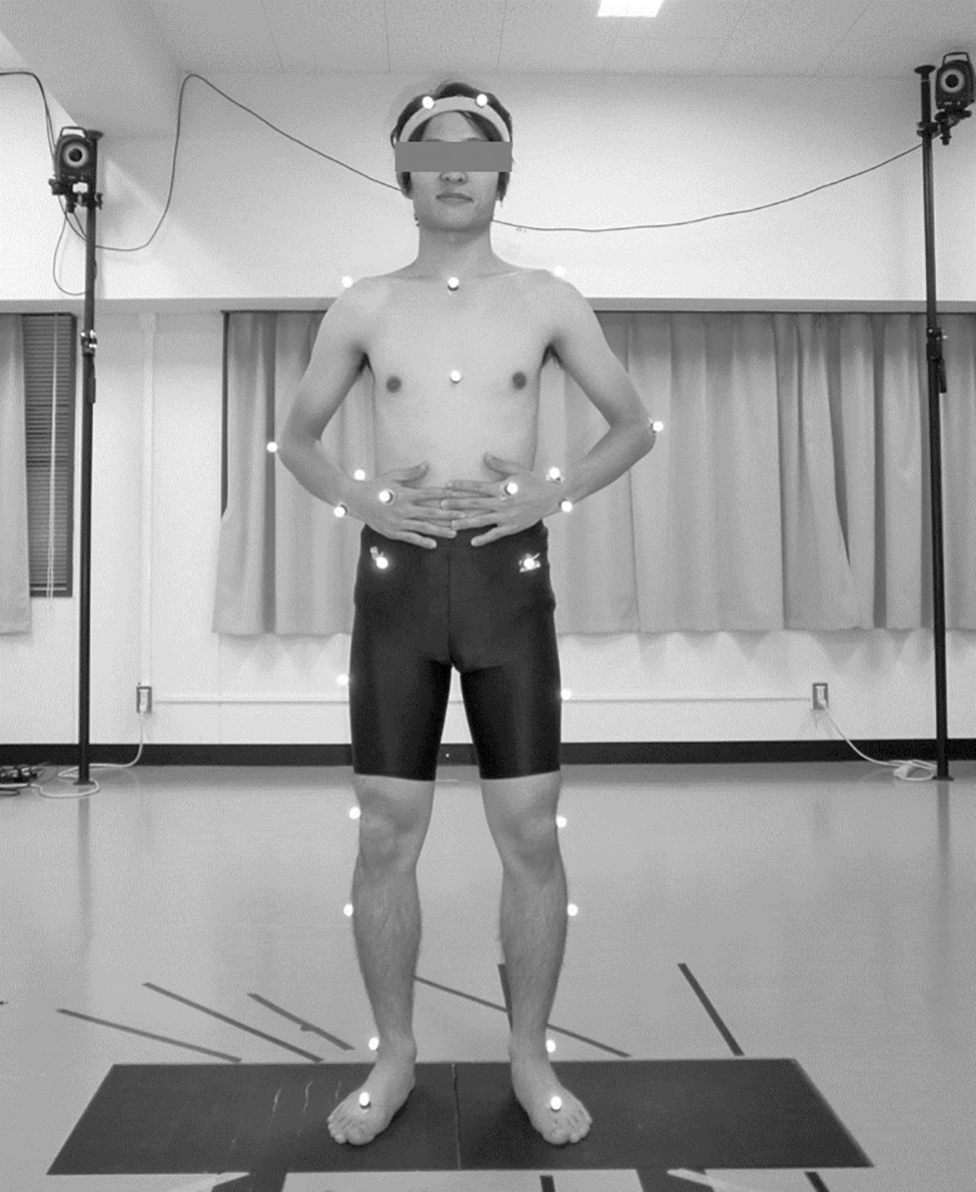
The starting position of motion task. The reflective markers were attached to specific placement on the subjects.

The data were collected with the subject in the standing position, with a toe-out angle of 10° and their hands on their abdomen. While standing, the distance between both calcanei was set equal to the individual’s foot length (Fig 1). Data were initially collected for 3 s in the standing position, followed by the examination of the kinematics of trunk rotation. The subjects were asked to trunk rotation from the neutral position as far as they could to left side, return to the initial position, rotate as far as they could to the opposite side, and finally return to the initial neutral position. The subjects performed each movement in 1 s, which means it took 4 s to complete the task. In order to adjust its effect on the motion task, the motion speed was controlled using a metronome. After ensuring that were no bilateral differences in ground reaction force between both the foot, in real time at the starting position, each trial was started as described previously [8]. Before experimental data collection, the subjects practiced the trunk rotation task at least three times to familiarize themselves with the motion speed. Measurements were repeated thrice.

### Procedures

Three-dimensional kinematics were recorded using a 6-camera Vicon motion system (Vicon Nexus; Oxford Metrics Ltd., Oxford, UK) at a sampling rate of 200 Hz, and a multicomponent force plate (type 9286A; Kistler, Switzerland) at a sampling rate of 1000 Hz. The subjects were clothed in close-fitting briefs, and reflective markers were attached to the body according to the Vicon Plug-in-Gait full-body model (Vicon; Oxford Metrics Ltd., Oxford, UK) marker placement protocol (Fig 1). The thoracic segment contained 6 markers positioned at the following sites: the seventh cervical and tenth thoracic vertebrae, jugular notch, xiphoid process of the sternum, and left and right acromioclavicular joints. The pelvic segment had 4 markers positioned at the following sites: the left and right anterior superior iliac spine and left and right posterior superior iliac spine [15]. The axis and direction were defined by the spatial coordinates prescribed in the laboratory. In the present study, the X-, Y-, and Z-axes represented the sagittal, frontal, and transverse planes, respectively. The angles of the thorax and pelvic segment were measured with reference to the global frame. Positive values represented anterior tilt, lateral flexion, and axial rotation to left side for the thorax and pelvis. We defined the three-dimensional kinematics of spinal motion to calculate the relative spatial changes between the thoracic and pelvic segment. Positive values are flexion and lateral flexion and axial rotation to left side for spine. All these procedures were carried out as described previously by Wada et al [16]. All data were low-pass filtered using a Woltring filter with a cut-off frequency of 6 Hz. Two subjects (a man in each group) were lost due to technical errors.

### Outcome measures

**Fig 2:**
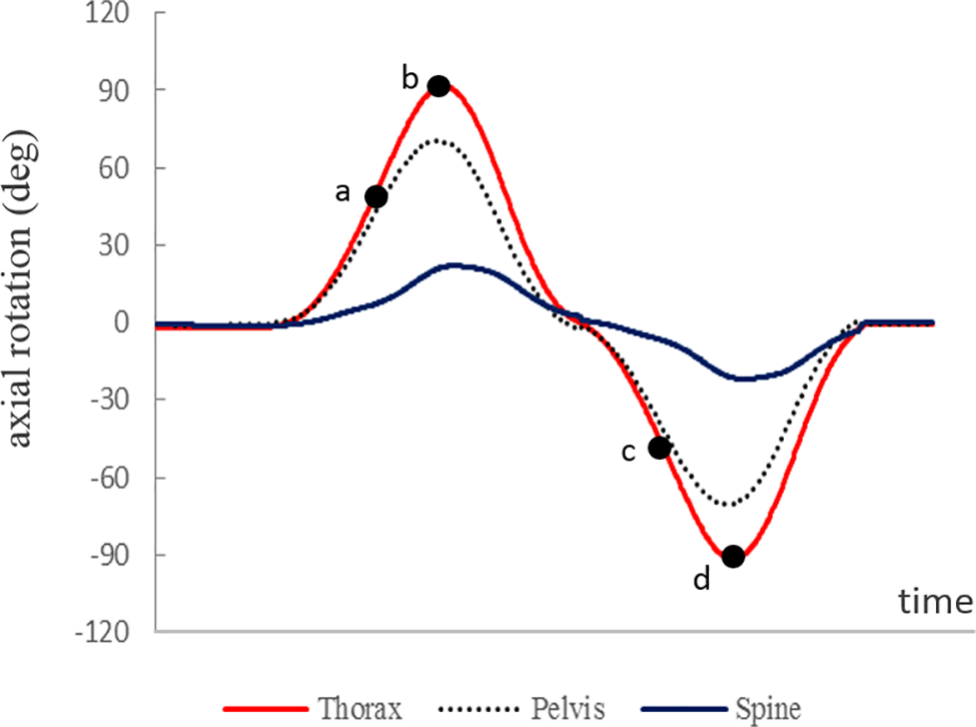
A representative curve of kinematic data during trunk rotation. Curves show the ROM of thorax (red line), pelvis (dotted line) and spine (blue line) in the transverse plane. The subjects performed trunk rotation task from the neutral standing position to the left, then to the right, and returned to neutral. The angles at 50% rotation position (a) and maximum position (b) during left side rotation, 50% rotation position (c) and maximum position (d) during right side rotation were measured. The positive value represents the phase during left side rotation, and the negative value represents the phase during right side rotation.

The angles of the thorax, pelvis, and spine in a static position were calculated. The primary outcomes measured were the rotational ROM and the spine/pelvis ratio (SPR) in transvers plane. Fig 2 shows a typical curve of kinematic data in the transverse plane during trunk rotation. The rotation ROM of the thorax, pelvis, and spine were measured at both maximum and 50% rotation position. The maximum rotation ROM was defined that summed the absolute values of left and right angle at maximum rotation position. Similarly, the 50% rotation ROM was defined that summed the absolute values of left and right at 50% rotation position relative to maximum. In addition, the SPR, which is defined as the ratio of the magnitude of the spine movement to that of the pelvis, was used as an index of movement pattern [6]. An important advantage of this method is that the SPR is normalized to each individual’s mobility during trunk rotation [17]. As secondary outcomes, the trunk rotation asymmetry (TRA) in transvers plane and the coupled angles in sagittal and frontal planes during trunk rotation were measured. TRA were calculated using the asymmetry index, which defines the bilateral difference between the maximum range to the left and to the right. We used the following equation: TRA = |(right rotation)—(left rotation)|/(right rotation) + (left rotation) × 100%. In addition, as a measurement of the coupled motion, the coupled angles in each segment were measured as the raw values in sagittal and frontal planes, at maximum rotation position. The coupled angles in the side with larger thorax rotation angle were used for data analysis. These outcome measures were processed using custom software (MatLab R2008b; The Mathworks Inc., Natick, MA).

### Statistics

All statistical tests were performed using SPSS statistical software (version 11.0; IBM Corporation, Armonk, NY). The subject characteristics were compared using student’s t-test for continuous variables with a normal distribution and the chi-square test for categorical variables. Before analyzed the primary outcomes, unpaired *t* tests performed to compare with a standing position. A two-way analysis of variance (ANOVA) was performed for rotation ROM of dependent variable to determine the interaction and main effects of group (CLBP and control) and segment (pelvis and spine). Unpaired *t* tests were applied to rotational ROM and SPR to determine the inter-group differences. Additionally, the TRA and coupled angles were compared between two groups using unpaired *t* tests. The alpha level for determining statistical significance was set at 0.05.

## Results

### The rotational ROM and SPR in transvers plane

**Fig 3:**
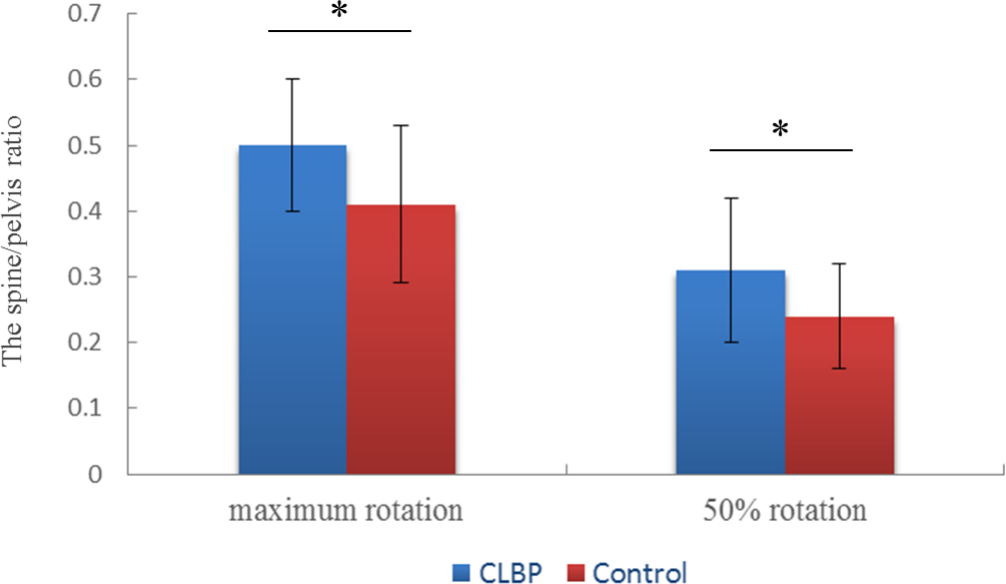
The SPR between CLBP patients and control. The spine/pelvis ratio (SPR) between CLBP patients and control at maximum and 50% trunk rotation. The higher SPR indicates relative hyper-mobility of spine regarding to pelvis. * indicates the statistical significance level of p<0.05.

No significant differences in the angles of the thorax, pelvis, and spine were found between the 2 groups in a static standing position (Table 2). There was a significant interaction between groups and segments (F = 4.57, p = 0.04, partial η^2^ = 0.08), but no significant main effects between groups (F = 0.40, p = 0.53, partial η^2^ = 0.01) at maximum rotation ROM. No significant differences were observed between the patients with CLBP and the healthy subjects with respect to the overall angles. The SPR was significantly greater in patients with CLBP than in the healthy subjects (Fig 3). The results of the rotational ROM and SPR in transvers plane are presented in Table 3.

**Table 2:** The angles of the thorax, pelvis, and spine in a static standing position.

	CLBP patients	Controls	*P*-value	95% CI
	Mean ± SD	Mean ± SD		
Sagittal plane				
Thorax (degree)	–4.2 ± 4.1	–1.7 ± 3.5	0.10	–5.50–0.47
Pelvis (degree)	14.1 ± 4.9	11.0 ± 7.5	0.22	–1.89–7.98
Spine (degree)	–18.3 ± 7.4	–12.7 ± 9.6	0.10	–12.23–1.12
Frontal plane				
Thorax (degree)	-0.1 ± 1.2	0.6 ± 2.9	0.37	–2.53–0.96
Pelvis (degree)	-0.3 ± 1.6	0.1 ± 2.0	0.54	–1.86–0.99
Spine (degree)	0.2 ± 1.9	0.6 ± 2.5	0.65	–2.07–1.32
Transverse plane				
Thorax (degree)	–1.4 ± 2.6	–1.3 ± 2.5	0.93	–2.09–1.91
Pelvis (degree)	–0.7 ± 2.8	0.6 ± 1.9	0.19	–3.09–0.64
Spine (degree)	–0.8 ± 2.7	–1.9 ± 2.6	0.27	–0.91–3.19

CLBP: chronic low back pain; SD: standard deviation; CI: confidence intervals; ROM: range of motion. Positive values represented anterior tilt for thorax and pelvis and flexion for spine in sagittal plane. Positive values represented elevation tilt for thorax and pelvis and lateral flexion for spine toward the left side in frontal plane.

**Table 3:** The rotational ROM and SPR in transvers plane.

	CLBP patients	Controls	*P-*value		95% CI
	Mean ± SD	Mean ± SD			
maximum rotation ROM					
Thorax (degree)	178.9 ± 21.6	183.8 ± 22.9	0.57		–12.45–22.19
Pelvis (degree)	120.1 ± 16.8	130.8 ± 15.8	0.09		–1.96–23.32
Spine (degree)	58.8 ± 10.5	53.0 ± 13.9	0.22		–15.37–3.76
SPR	0.50 ± 0.10	0.41 ± 0.12	0.04	^*^	–0.17 - –0.01
50% rotation ROM					
Thorax (degree)	88.2 ± 11.1	90.0 ± 11.5	0.69		–7.03–10.52
Pelvis (degree)	67.5 ± 8.9	72.9 ± 8.5	0.11		–1.36–12.16
Spine (degree)	20.8 ± 7.0	17.1 ± 6.0	0.15		–8.71–1.41
SPR	0.31 ± 0.11	0.24 ± 0.08	0.04	^*^	–0.15 - –0.01

CLBP: chronic low back pain; SD: standard deviation; CI: confidence intervals; ROM: range of motion; SPR: spine/pelvis ratio.

*A *P*-value <0.05 on the unpaired *t* test indicates a significant difference between the 2 groups.

There was also a significant interaction between groups and segments (F = 4.85, p = 0.03, partial η^2^ = 0.09), but no significant main effects between groups (F = 0.18, p = 0.67, partial η^2^ = 0.00) at 50% rotation ROM during trunk rotation. In similar with the results in maximum rotation ROM, no significant differences were observed between the 2 groups with respect to the overall angles. However, the SPR was significantly greater in patients with CLBP, compared with the healthy subjects (Table 3 and Fig 3).

### The trunk rotation asymmetry in transvers plane

There were no significant differences between the 2 groups with respect to the thorax (CLBP patients; 3.8 ± 3.4%, Controls; 3.7 ± 2.7%, p = 0.97), pelvis (CLBP patients; 3.6 ± 2.1%, Controls; 4.6 ± 4.4%, p = 0.43), and spine (CLBP patients; 10.9 ± 9.3%, Controls; 7.5 ± 5.3%, p = 0.26) in the asymmetry index.

### Coupled motion in sagittal and frontal planes

Table 4 shows the results of coupled motion in sagittal and frontal planes. CLBP patients exhibited a significant difference in the sagittal plane coupled with trunk rotation. The anterior tilt of the pelvic segment and spine extension in patients with CLBP were greater than those of the healthy subjects. Coupled motion in the lateral tilt and/or lateral flexion of the pelvis and spine showed no significant differences in the frontal plane between the 2 groups.

**Table 4:** Coupled motion in sagittal and frontal planes.

	CLBP patients	Controls	*P*-value		95% CI
	Mean ± SD	Mean ± SD			
Sagittal plane					
Thorax (degree)	0.6 ± 2.6	3.4 ± 3.8	0.04	^*^	0.18–5.26
Pelvis (degree)	14.7 ± 4.8	10.8 ± 3.9	0.03	^*^	–7.30 - –0.48
Spine (degree)	-14.1 ± 6.2	-7.4 ± 6.1	0.01	^*^	1.93–11.42
Frontal plane					
Thorax (degree)	-5.3 ± 4.1	-2.5 ± 1.4	0.02	^*^	0.44–5.20
Pelvis (degree)	7.5 ± 7.6	5.0 ± 6.6	0.36		–7.96–3.03
Spine (degree)	-12.8 ± 10.3	-7.5 ± 7.2	0.13		–1.60–12.19

CLBP: chronic low back pain; SD: standard deviation; CI: confidence intervals; ROM: range of motion. Positive values represented anterior tilt for thorax and pelvis and flexion for spine in sagittal plane. Positive values represented elevation tilt for thorax and pelvis and lateral flexion for spine toward the left side in frontal plane.

*A *P-*value <0.05 on the unpaired *t* test indicates a significant inter-group difference.

## Discussion

The results of present study indicated that there were no significant differences in the rotational ROM of the thorax, pelvis, and spine, between the patients with CLBP and the healthy subjects, during trunk rotation. However, there was a significant interaction between groups and rotational ROM of pelvis and spine, and the SPR in patients with CLBP was significantly greater than that of the healthy subjects. These results support our hypothesis that kinematics characteristics in patients with CLBP differ in the movement pattern of the pelvis and spine, not but in maximum rotation ROM in transverse plane compared with healthy subjects. The coupled motion in sagittal plane, which accompanies trunk rotation, were greater than that of the healthy subjects; and these results further support our hypothesis.

Sung et al. [8] have reported that the rotational ROM of upper thorax in CLBP patients was significantly lower, and that of lumber was greater, than control group during maximum trunk rotation. However, our results in the rotational ROM were not consistent with the previous study. Since the spinal ROM in this study was defined as the relative spatial changes between the thoracic and pelvic segment, the disparity in results may be due to the difference in the three-dimensional model employed. The SPR was significantly greater in the patients with CLBP than in the healthy subjects because of the movement pattern during trunk rotation. The previous study also indicated an increase in the relative mobility of spine [8], which was similar to our results. An increase in the relative mobility of spine was found at both maximum and 50% rotation ROM. Therefore, these results suggest that CLBP patients had different movement pattern compared with healthy subjects not only at the end rage of trunk rotation. As trunk rotation was achieved by the high contribution of rotational ROM in the pelvis [6], the relative decrease in pelvic mobility may be associated with the compensatory movement pattern by spine mobility. Sahrmann et al., [18] defined the phenomenon in which a joint with high flexibility begins to move primarily (as compensatory relative flexibility), which can be seen when various joints move in the same direction, such as in trunk rotation. Actually, previous studies have reported that CLBP patients have an abnormal relative timing and asymmetry of pelvic and hip rotation [19, 20]. Therefore, uncoordinated movement pattern such as the increase of SPR in patients with CLBP has suggested to associate with the mobility of pelvic segment. Previous study [21] have reported that CLBP patients had less pelvic and unchanged thoracic rotation during walking as compared to healthy controls. Although the motion tasks were different, their results suggested a relative increase in spinal rotation, consistent with the results of present study. Another study [22] showed asymmetry pattern in spine motion in CLBP patients during walking, therefore these findings suggested that CLBP patients had uncoordinated movement patterns during walking as well as during trunk rotation.

The results of present study that the CLBP patients had significantly greater motion of the pelvis and spine in the sagittal plane coupled with trunk rotation were consistent with previous study [9]. However, considering the lower limits of the 95% confidence intervals, the pelvic anterior tilt angle showed only a minor difference in patients with CLBP, which may not be of clinical importance. By contrast, the spinal extension angle in patients with CLBP was greater than that of control subjects, with a satisfactory difference in the 95% confidence intervals. Hyper rotation in the lumbar vertebral segment itself does not induce intervertebral disc injury, but compounded movements in other planes can increase the risk of LBP [12, 23]. Thus, the increased mechanical stress of the spine in CLBP patients may be associated with excessive spinal extension coupled with trunk rotation, in addition to abnormal movement pattern in the transverse plane. Although the intra-abdominal pressure (IAP) increase stiffness of the lumber spine [24, 25], trunk rotation was disadvantageous for elevating the IAP [26]. Furthermore, muscle activity of multifidus muscle which contributes to spinal stabilization was decreased in CLBP patients during trunk rotation [27]. Spinal instability due to decrease in IAP and muscle activity may be related to excessive spinal extension coupled with trunk rotation.

This study has several limitations. Firstly, the three-dimensional model used in present study should be assessed with caution because the thorax was considered a single rigid segment based on the markers set on VICON model. However, the thorax consists of several mobile parts. To simplify the analysis, it is generally assumed that the thorax is a rigid segment [28]. Although more detailed trunk models provide more detailed spinal motion information, it is difficult to evaluate trunk motion using these models in the clinical setting. Since the VICON model has been widely used in several studies [16, 29, 30], it can be judiciously used for the assessment of spinal motion. The second limitation was the selection of the CLBP patients, which included various types of back pain caused by numerous factors (for example, trunk forward and backward bending, axial rotation, and standing for prolonged time). Therefore, the motion task in this study may not be a critical movement for each CLBP patients. It is necessary to classify the CLBP subjects into subgroups based on the painful motion and to analyze kinematics in future studies.

## Conclusions

The present study showed that patients with CLBP had relative hyper rotational mobility of the spine as well as excessive spinal extension coupled with trunk rotation. The results suggest that uncoordinated movement during trunk rotation might be a functional failure associated with CLBP.

## Supporting information

S1 Table Low back pain questionnaire. The subjects who experience low back pain with an intensity greater than 30 mm on the visual analog scale and with duration of >3 months, kindly answer this questionnaire.
(DOCX)

S2 Table Low back pain questionnaire (Japanese Edition). The subjects who experience low back pain with an intensity greater than 30 mm on the visual analog scale and with duration of >3 months, kindly answer this questionnaire in Japanese.
(DOCX)
