# Supplementary material for: Relative mobility of the pelvis and spine during trunk axial rotation in chronic low back pain patients: A case-control study
Source: PLoS One. 2017 Oct 17;12(10):e0186369. doi: 10.1371/journal.pone.0186369 (PMC5645112; doi:10.1371/journal.pone.0186369)
Supplement: S1 Table — The subjects who experience low back pain with an intensity greater than 30 mm on the visual analog scale and with duration of >3 months, kindly answer this questionnaire. (DOCX) [file pone.0186369.s001.docx]

**LBP check list**

Name：　　　　　　　Data：　　　.　　.

Orthopaedic surgeon：

Investigator：

＊The subjects who experience low back pain with an intensity greater than 30 mm on the visual analog scale (VAS) and with duration of >3 months, kindly answer this questionnaire.

1. **About your low back pain (LBP)**

a. When did your LBP occur? Since when do you have LBP?

b. How much pain did you experienced?

No pain The strongest pain you have ever experienced.

c. Do you have a probable cause for LBP?

d. What kind of motion task induces the LBP? (Multiple answers possible)

forward bending　lateral bending　twisting　standing　sitting　　　lying on

e. Have you sought any medical attention for your LBP? (Yes / No)

→ If Yes, please fill in as much detail as possible following.

Diagnosis:

　　　About doctor’s explanation and treatment contents.

(　　　　　　　　　　　　　　　　　　)

　　→If No, please check the items as follows.

1. Do you have numbness from lower back to legs, when you bend forward? (Yes / No)
2. Do you have numbness from lower back to legs, when you dorsiflex the ankle? 　(Yes / No)
3. Do you have numbness from lower back to legs, when you bend laterally?　　　 (Yes / No)
4. Do you have numbness from lower back to legs, when you extend backward?　 (Yes / No)
5. Do you have low back pain associated with fever, anemia, or abdominal pain? (Yes / No)
6. Do you have low back pain such as acute strained back? (Yes / No)
7. Is your pain relieved by taking break, or do you feel numbness while walking? (Yes / No)
8. **Have you sought medical attention for any urological diseases? (Yes / No)**

→ If Yes, please fill in as much detail as possible.

Diagnosis:

　　About doctor’s explanation and treatment contents.

(　　　　　　　　　　　　　　　　　　)

1. **Have you sought medical attention for any gastrointestinal diseases? (Yes / No)**

→ If Yes, please fill in as much detail as possible following.

Diagnosis:

　About doctor’s explanation and treatment contents.

(　　　　　　　　　　　　　　　　　　)
