# Supplementary material for: Relative mobility of the pelvis and spine during trunk axial rotation in chronic low back pain patients: A case-control study
Source: PLoS One. 2017 Oct 17;12(10):e0186369. doi: 10.1371/journal.pone.0186369 (PMC5645112; doi:10.1371/journal.pone.0186369)
Supplement: S2 Table — The subjects who experience low back pain with an intensity greater than 30 mm on the visual analog scale and with duration of >3 months, kindly answer this questionnaire in Japanese. (DOCX) [file pone.0186369.s002.docx]

**腰痛チェック表**

名前：　　　　　　　記入日：H　　.　　.

医師：

説明・記入者：

＊過去にVAS30mm以上の痛みが3カ月以上続く腰痛を経験した方のみご記入ください。

1. **あなたの腰痛についてお尋ねします。**

a.いつ頃から痛くなりましたか？その痛みは、どのくらいの期間続きましたか？

b.どの程度の痛みでしたか？

　　

c.痛みが出たきっかけや原因はありました？

d.どのような動作で痛みが出現しましたか？(複数回答可）

前屈したときに痛い　　身体を横に倒した時に痛い　　身体を回した時に痛い

立っていると痛い　　　座っていると痛い　　　　　　寝ていても痛い

e.痛みが生じた際、医療機関を受診しましたか？　(　Yes　/　No　)

→Yesの場合には、以下に可能な限り詳細に記入してください。

診断名：

　　　医師からの説明や治療内容について

(　　　　　　　　　　　　　　　　　　)

　　→Noの場合には、以下の質問にお答えください。

1. 身体を前屈した時、腰から足にかけてしびれが生じた　　　　　　　(　Yes　/　No　)
2. 足首を上に向けると、腰から足にかけてしびれが生じた　　　　　　(　Yes　/　No　)
3. 身体を横に倒した時、腰から足にかけてしびれが生じた　　　　　　(　Yes　/　No　)
4. 身体を後ろにそらすと、腰から足にかけてしびれが生じた　　　　　(　Yes　/　No　)
5. 腰痛の他、発熱や貧血、腹痛などを伴った痛みが生じた　　　　　　(　Yes　/　No　)
6. 瞬時に発症したが、日ごとに軽減し、腰痛が消失した　　　　　　　(　Yes　/　No　)
7. 歩行時、足にしびれが生じて歩行が困難になるが、休憩で回復した　(　Yes　/　No　)
8. **今までに泌尿器系疾患で診察・治療を受けたことがありますか？ (　Yes　/　No　)**

→ Yesの場合には、以下に可能な限り詳細に記入してください。

　　診断名：

　　症状や期間、その後の治療や現在の状態について

(　　　　　　　　　　　　　　　　　　)

1. **今までに消化器系疾患で診察・治療を受けたことがありますか？ (　Yes　/　No　)**

　→ Yesの場合には、以下に可能な限り詳細に記入してください。

　　診断名：

　　症状や期間、その後の治療や現在の状態について

(　　　　　　　　　　　　　　　　　　)
